# An Analytical Method for Mechanical Analysis of Offshore Pipelines during Lifting Operation

**DOI:** 10.3390/ma16206685

**Published:** 2023-10-13

**Authors:** Zhongxiao Wang, Yongxin Chen, Qingyou Gao, Fengming Li

**Affiliations:** 1 College of Aerospace and Civil Engineering, Harbin Engineering University, Harbin 150001, China; wangzxwork99@163.com; 2 Offshore Oil Engineering Co., Ltd., Tianjin 300461, China; chenyx22@cooec.com.cn (Y.C.); gaoqy2@cooec.com.cn (Q.G.)

**Keywords:** lifting of offshore pipelines, mechanical property, analytical method, finite element method, Orcaflex

## Abstract

The lifting operation of offshore pipelines is an important step in ocean pipeline engineering. An effective analytical method is developed for investigating the mechanical properties of the pipeline based on mechanical, physical, and geometric relationships. By using the shooting and the secant methods to transform the boundary value problem into an initial value one and then solving them with the Runge–Kutta method, the deformation and mechanical properties of the pipeline are calculated. Furthermore, based on the Det Norske Veritas (DNV) offshore standard, the mechanical properties of the pipeline are checked. The finite element method (FEM) by Orcaflex is employed to verify the accuracy of the analytical model. The effects of some factors such as the current velocity and lifting point position on the mechanical properties of the pipeline are analyzed based on the analytical model. The results indicate that the change in current velocity during the lifting process has a minimal effect on the pipeline, but the change in lifting point position significantly affects the deformation and mechanical properties of the pipeline.

## 1. Introduction

As population expands, the environment deteriorates, and resources become scarce, the ocean demonstrates clear advantages in terms of space, resources, environment, and strategy due to continuous advancements in science and technology. Ocean resources, including fisheries, space, and energy, have become an area with tremendous potential for development in the twenty-first century [1,2]. Oil and gas resources, which are currently the main focus of utilization, have led countries around the world to actively engage in exploration and development [3,4]. In the process of extracting, refining, storing, and transporting offshore oil and gas resources to end-users, pipelines play a crucial role as a convenient, safe, cost-effective, and reliable means, tightly connecting the entire production process of marine resource development [5,6,7] and serving as a vital element in ensuring the smooth operation of resource development.

Many researchers and engineers use numerical calculation tools such as Orcaflex v11.0 [8,9,10,11,12], ABAQUS v2020 [13,14,15,16], and ANSYS v10.0 [17,18] to conduct research on offshore pipelines. Wang et al. [12] simulated different stages of a drilling pipe and subsea manifold in Orcaflex, investigating the effects of current velocity, wave height, and bundle weight on tension, bending moment, stress, and displacement of the pipe. The oscillation of the subsea manifold is easily affected by wave height in the splash zone. The displacement increases with an increase in current velocity but decreases with an increase in manifold weight while descending underwater. When the manifold is installed on the seabed, the bending moment and stress at the top of the drilling pipe increase with increases in current velocity and wave height, while at the seabed, it mainly increases as the current velocity increases. Zhang et al. [17] used ANSYS to calculate the impact of submarine landslides on laid or suspended offshore pipelines at different impact angles. Equations for calculating the axial and normal drag coefficients of a submarine pipeline were presented. Rao and Kaliveeran [18] employed both ANSYS and an experimental method to study the effects of the sizes of buckle arrestors and their positions along a length of pipeline on the structural performance of offshore pipeline. The buckling capacity of the pipeline can be improved up to 50% and 250% using rectangular pin stiffeners at the midpoint of the pipeline and longitudinal continuous stiffeners.

Experimental testing methods [19,20,21,22,23] are also applied to research on offshore pipelines. Liang et al. [20] conducted experiments to study the dynamic interaction between pipelines and stabbing rollers during pipeline installation. The experiments indicated that the heave had a more significant influence on the dynamic roller force than the pitch and roll. However, using numerical simulation tools for calculations is time-consuming, and the steps are complex, making it difficult to derive the basic principles. Additionally, due to high costs and technical limitations of laboratory facilities, physical modeling experiments are usually limited to partial or small-scale models. Therefore, in the initial stages of ocean pipeline design and construction, engineers tend to seek simple and time-saving methods to complete preliminary analysis. Hence, the analysis of ocean pipeline construction requires simple, effective, and reasonable theoretical calculation models. Song et al. [24] established an indirect time-domain coupling dynamic model of a vessel-lifting pipe by combining the analytical methods of vessels and lifting pipes. The results show that the coupling effect has a significant impact on the vessel–pipe dynamic behavior.

The lifting of damaged underwater pipelines to the water surface for repair or installation of integrated risers involves lifting operations. During the lifting process, one side of the pipeline is placed on the seabed while the other side is suspended. This requires a long suspension span and results in significant bending and deformation of the pipeline, leading to complex mechanical properties. Therefore, it is necessary to analyze the mechanical properties of the pipeline during the lifting process to ensure that there is no damage during pipeline-lifting operations and the construction can proceed safely. Scholars have proposed theoretical calculation methods for the mechanical properties of pipelines. Plunkett [25] proposed the catenary method which modeled a pipeline and used the asymptotic expansion method to solve a large-deflection nonlinear problem wherein the effect of tension was greater than that of bending rigidity. Dareing and Neathery [26] derived a linear differential equation using Newton’s method to address the large-angle bending problem of deep-sea pipes which cannot be fully defined by small deformation theory. The finite difference method was then used for numerical solutions. Pedersen [27] established the control equation considering bending rigidity, wet weight, and ocean current, and non-dimensionalized the nonlinear boundary value problem. Continuous integration was used for numerical solutions. Datta and Basu [28] established a fourth-order nonlinear differential equation, considering wet weight and the forces applied by barges as the causes of pipeline deformation. An approximate solution was obtained using the Newton–Raphson method combined with the Jacobian matrix.

Based on the finite difference method proposed by Datta and Basu [28], Andreuzzi and Maier [29] further considered the influence of ocean current and conducted an analysis on the suspended part of a pipeline. The assumed projection length between the two ends of the pipeline was obtained, and the problem was transformed into a regular boundary value problem. Guarracino and Mallardo [30] used the singular perturbation method for analytical solutions and compared the results with finite element analysis. On the basis of previous research, subsequent studies delved into specific issues. Lenci and Callegari [31] focused on the J-lay pipe-laying problem and improved upon the classical catenary theory by constructing four models to qualitatively and quantitatively verify the significance of boundary conditions. Cheng and Polak [32] developed a computational model to calculate the mechanical properties of pipelines being pulled during horizontal directional drilling. The analysis considered the effects of directional changes, fluid resistance, solid friction, and pipeline weight on the load borne by the pipeline. Szczotka [33] used a rigid finite element method (FEM) to simulate the installation and laying process of the pipeline with a computational time reduction of 65–70% compared with traditional finite element simulations.

Ruan et al. [34] established an analysis model for the mechanical behavior of deep-water pipelines considering environmental loads and the elastic effect of the seabed. The relationship between seabed stiffness, tilt angle of suspension, and segment length of buoyancy was analyzed. Trapper [35] treated the entire pipeline, including both suspended and seabed-laid ends, as a continuous segment and proposed a numerical method for structural analysis of the pipeline considering both S-lay and J-lay configurations, using the minimum potential energy principle and the finite difference method. Furthermore, the effects of laying angle, tension, water depth, and seabed stiffness on the configuration and mechanical performance of the pipeline during the laying process were investigated. Xu et al. [36] combined the principles of mechanical vectors with a numerical algorithm based on a vector form of FEM. The analysis considered internal forces induced by element deformation and node rotation as well as external forces caused by hydrodynamic loads and boundary interactions during the laying process. Li et al. [37] used the vector form intrinsic FEM to analyze the nonlinear behavior of marine risers with large deformations in three-dimensional space. It was proven that the application of the vector form intrinsic FEM in the nonlinear analysis of the three-dimensional marine risers is feasible. In addition, in recent years, many further investigation results have been published in this regard [38,39,40,41,42,43].

The deformations of submarine pipelines during lifting and lowering processes are essentially large displacements, falling under the category of geometrically nonlinear large deformation problems. Additionally, due to the stiffness of large-diameter pipelines, the bending moment generated during the pipeline deformation process cannot be neglected. In this paper, the suspended section of a pipeline during the lifting process is researched. By establishing control equations based on force balance, physical relationships, and geometric relationships along the axial and transverse directions of the pipeline, an effective theoretical calculation model to determine the spatial configuration and mechanical performance of the pipeline during the lifting process is developed. Based on the obtained mechanical property and DNV standards [44], the mechanical performance of the pipeline is further evaluated. Finally, using this model, the impact of the current velocity and the positions of lifting points on the mechanical properties of the offshore pipeline during the lifting process is investigated.

The aim of this study is to develop an effective theoretical calculation model to determine the spatial configuration and mechanical performance of an offshore pipeline during the lifting process and to provide engineers with a simple and time-saving method to complete preliminary analysis in the initial stages of offshore pipeline design and construction.

## 2. Theoretical Model

### 2.1. Model of Suspended Section of Pipeline

**Figure 1:**
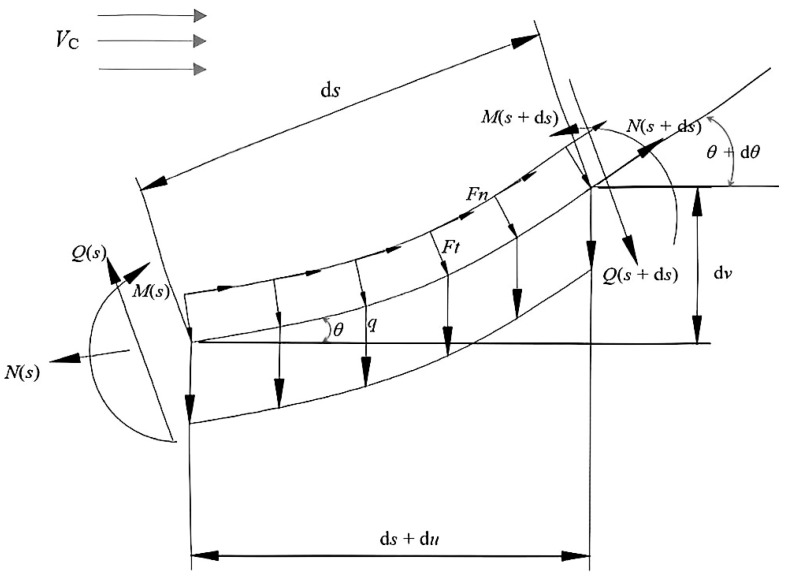
The model of the suspended section of pipeline in the sea.

A model diagram of the suspended section of pipeline is displayed in Figure 1. The length of the element is d*s*, and *V_c_* is the velocity of the ocean current.

The force equilibrium equation of the suspended section of pipeline is given by the following equations:

(1) −N−qdssinθ+(N+dN)cosdθ+(Q+dQ)sindθ+Ftds=0,

(2) Q−qdscosθ−(Q+dQ)cosdθ+(N+dN)sindθ−Fnds=0,

(3) $$-M-qds\cos\theta \frac{ds}{2}-(Q+dQ)\cos d\theta ds+(M+dM)+(N+dN)\sin d\theta ds-Fn\frac{d^{2}s}{2}+Ft\frac{d^{2}s}{2}\tan\theta =0,$$

where *q* is the gravity of unit length of the pipeline in the sea, *N* and *Q* are the axial and shear forces in the section of pipeline, *F_t_* and *F_n_* are the environmental loadings in the section of pipeline, *M* is the bending moment of the section of pipeline, and *θ* is the inclination angle of the section of pipeline.

Due to d*s* → 0 and d*θ* → 0, cosd*θ* → 1, sind*θ* → d*θ*, d*θ*d*s* → 0, and d*s*d*s* → 0. Equations (1)–(3) can be simplified as

(4) −qdssinθ+dN+Qdθ+Ftds=0,

(5) −qdscosθ−dQ+Ndθ−Fnds=0,

(6) dM−Qds=0.

Axial and shear deformations are ignored, so the physical equation is established considering bending deformation as

(7) $$EI\frac{d\theta }{ds}=M,$$

where *EI* is the flexural rigidity of the pipeline.

The geometric equation is given by

(8) $$\sin\theta =\frac{dv}{ds},$$

(9) $$\cos\theta =\frac{ds+du}{ds}=1+\frac{du}{ds},$$

where *v* and *u* are the transverse and axial displacements of the pipeline section.

For convenience, the following dimensionless variables are introduced:

(10) $$c=\frac{s}{a},\;v(c)=\frac{v(s)}{a},\;u(c)=\frac{u(s)}{a},\;M(c)=\frac{M(s)}{aq^{2}},\;\theta (c)=\theta (s),\;N(c)=\frac{N(s)}{aq},\;Q(c)=\frac{Q(s)}{aq},$$

where *a* is the length of the suspended section of the pipeline.

Considering the dimensionless variables in Equation (10), the simplified governing equations of the section of pipeline, Equations (4)–(9), can be further expressed in the following dimensionless forms:

(11) $$-\sin\theta +\frac{dN}{dc}+Q\frac{d\theta }{dc}+\frac{Ft}{q}=0,$$

(12) $$-\cos\theta -\frac{dQ}{dc}+N\frac{d\theta }{dc}-\frac{Fn}{q}=0,$$

(13) $$\frac{dM}{dc}=Q,$$

(14) $$\frac{d\theta }{dc}=M\frac{a^{3}q}{EI},$$

(15) $$\sin\theta =\frac{dv}{dc},$$

(16) $$\cos\theta =1+\frac{du}{dc}.$$

The part of the pipeline that touches the seabed is assumed to be infinitely long, and the touchdown point (TDP) is regarded as the horizontal plane. The stiffness of the seabed has minimal impact on the actual construction process, since the contact area is very small compared to the relative increase in length [31,34]. The stiffness of the seabed making contact at the TDP is infinite. The support reaction of the seabed is simplified as the concentrated force at the TDP of the pipeline, and the reaction couple caused by the contact area is ignored. The TDP can be simplified as a hinge support with zero bending moment and only shearing force [28]. In this way, the TDP can be simplified into a hinge support with only shearing force and zero bending moment. The mechanical model of the suspended pipeline is represented by the six-element and first-order differential equations derived above. The deflection, horizontal displacement, angle, axial force, and bending moment at the TDP of the suspended pipeline model are zero, while the shear force is unknown and the bending moment at the rightmost end is determined according to construction conditions.

### 2.2. Environmental Loadings

**Figure 2:**
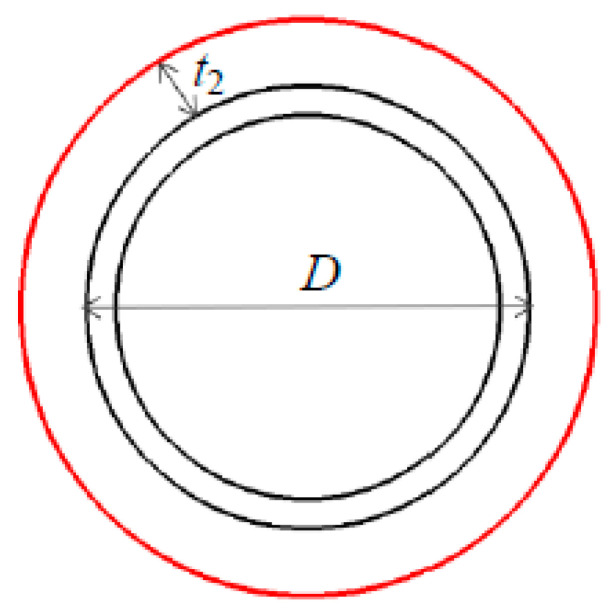
The schematic diagram of the cross section of the pipeline.

In the process of submarine pipeline construction, considering the currents of marine environments, the submarine pipeline mainly bears the action of seawater buoyancy and ocean currents. During the construction of the submarine pipeline, there are two states inside the pipeline: aeration and liquid filling. The contents are different, and the action of the submarine pipeline is different. However, under normal conditions, the inside of the pipeline is in an aerated state, and the buoyancy force received by the submarine pipeline is a uniformly distributed load directed vertically upward. The schematic diagram of the cross section of the pipeline is shown in Figure 2.

The expression of the uniformly distributed buoyancy load *w_b_* acting on the unit length of the section of pipeline is

(17) $$w_{b}=\frac{1}{4}\rho_{s}g\pi {\left(D+2t_{2}\right)}^{2},$$

where $\rho_{s}$ is the density of seawater, $t_{2}$ is the thickness of the coating layer, and *D* is the outer diameter of the steel part of the pipeline. In the calculations, the gravity of the pipeline in the sea *q* is obtained by the weight *w* of the unit length of the section of pipeline and the buoyancy *w_b_*.

The Morison formula is used to establish the relationship between ocean current velocity and the environmental loads acting on the submarine pipeline. With Morison’s equation [45,46,47,48], the ocean current-induced forces on the submarine pipeline in the normal (*F_n_*) and tangential (*F_t_*) directions can be written as

(18) $$F_{n}=\frac{1}{2}\rho_{s}C_{n}(D+2t_{2})\left|V_{c}\sin\theta \right|V_{c}\sin\theta ,$$

(19) $$F_{t}=\frac{1}{2}\rho_{s}C_{t}(D+2t_{2})\pi \left|V_{c}\cos\theta \right|V_{c}\cos\theta ,$$

where *C_n_* and *C_t_* are the normal and tangential drag coefficients, and *V_c_* is the velocity of ocean current.

### 2.3. Configuration Calculation

**Figure 3:**
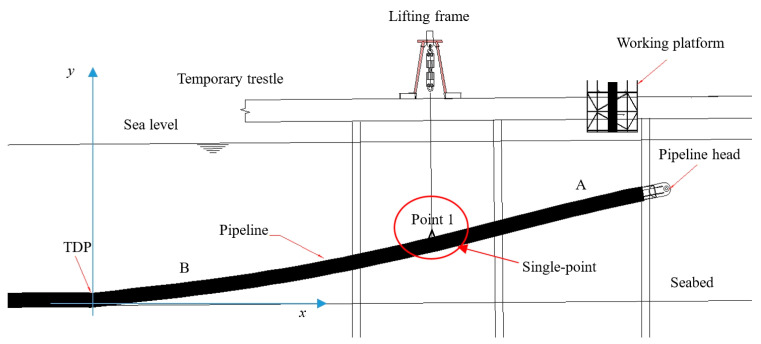
The sketch of single-point submarine pipeline suspension.

In the case of single-point submarine pipeline suspension as shown in Figure 3, the pipeline is divided into two parts, A and B, based on the position of the lifting point. Firstly, pipeline section A is treated as a cantilever beam. Since the bending moment of the entire pipeline remains continuous, the initial bending moment *M_A_* (start) of section A is equal to the final bending moment *M_B_* (end) of section B. Subsequently, the calculation of section B is performed. Considering the aforementioned assumptions regarding boundary conditions, the deflection, horizontal displacement, angle, axial force, and bending moment at the TDP of the suspended pipeline model are known, and the final bending moment of section B is also known. During the actual calculation process, the shear force *Q_B_* (start) at the TDP is considered as the shooting parameter. By employing the shooting method to adjust the initial shear force, the bending moment at the end of pipeline B can satisfy the condition *M_A_* (start), thereby transforming the boundary value problem into an initial value one for resolution.

Set the lifting length of section B to be *a*. After dimensionless treatment of differential equations, transform the solution range from 0–*a* to 0–1. Taking *Q_B_* (start) to *Q_B_* (0) as the shooting parameter, constantly adjust the value of *Q_B_* (0) to ensure that the pipeline head meets the boundary condition *M_B_* (1) = *M_A_* (0). By doing so, the dimensionless result of determining the pipeline under conditions of length *a* of the lifting section B can be solved.

The boundary value problem is converted into an initial value one using the secant method, which iteratively adjusts the shooting parameters. The specific calculation method is as follows: Firstly, two initial values *Q*_0_ (0) and *Q*_1_ (0) are selected for iterative calculation. Then, the corresponding final values *M*_0_ (1) and *M*_1_ (1) are calculated using the Runge–Kutta Method. Finally, the obtained initial and final values are substituted into the following equation to obtain the new shooting parameter *Q_m_* (0):

(20) $$Q_{m+1}(0)=Q_{m}(0)-\frac{M_{m}(1)-M_{A}(0)}{M_{m}(1)-M_{m-1}(1)}[Q_{m}(0)-Q_{m-1}(0)],\;(m=1,2\ldots ).$$

New shooting parameters are obtained from the previous calculations and used again in the above steps for recalculation until *M_m_* (1) − *M_A_* (0) becomes sufficiently small. This allows us to obtain an approximate numerical solution of the equation, where *Q_m_* (0) represents the contact shear force *Q_B_* (0). Finally, by utilizing the values of deflection, horizontal displacement, angle, axial force, bending moment, and shear force *Q_B_* (0) at the TDP, the deformation and mechanical properties of the entire pipeline in the case of single-point submarine pipeline suspension can be determined by solving Equations (11)–(16).

**Figure 4:**
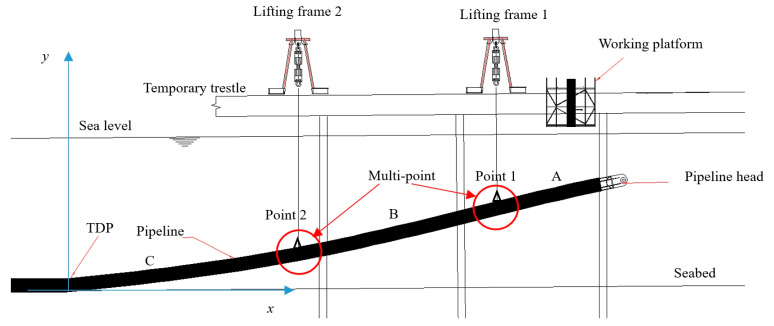
The sketch of multi-point submarine pipeline suspension.

In the case of multi-point submarine pipeline suspension as illustrated in Figure 4, the pipeline is divided into three parts, A, B, and C, based on the positions of two lifting points. Sections A and B are considered as a single entity due to the continuity of the bending moment across the entire pipeline. The initial bending moment *M_AB_* (start) of section AB is equal to the final bending moment *M_C_* (end) of section C. The parameters relevant to the pipeline in section C, between the TDP and point 2, are solved using the same method as the single-point suspension approach. Section A is treated as a cantilever beam, where the initial bending moment *M_A_* (start) of section A is equal to the final bending moment *M_B_* (end) of section B. The initial conditions of section B can be calculated from the end of section C, while the final conditions of section B can be calculated from the start of section A. By considering *Q_B_* (0) as the shooting parameter, the boundary value problem is transformed into an initial value problem using the secant method and the shooting method, as introduced in the single-point suspension approach, to solve section B. Finally, by using the same calculation method as applied in the case of single-point submarine pipeline suspension, the deformation and mechanical properties of the entire pipeline can be determined by solving Equations (11)–(16).

### 2.4. Check for Mechanical Properties and Engineering Parameters

#### 2.4.1. Von Mises Stress

Pipeline stress is a significant mechanical property parameter during offshore pipeline construction [49,50,51]. In severe cases, high stress can lead to plastic deformation and even fracturing of the pipeline. Stress calculation assumes that all loads at each point of the pipeline are applied to a cylinder with uniform material properties and specified stress on the outer and inner diameters. This stress consists of tension, bending, shearing, and hoop stresses. The wall tension is produced by the pressure inside the pipe, and the shear stress is assumed to be evenly distributed throughout the cross section. The calculation of circumferential stress does not consider pressure changes caused by flow fluctuations in the pipeline. While these assumptions are suitable for homogeneous pipes with high bending stiffness, such as steel, they are not applicable to composite flexible risers and rope chains. In engineering, the von Mises stress is commonly used to represent the mechanical properties of pipelines, and its calculation formula is written as

(21) $$\sigma_{vm}=\sqrt{\frac{{\left(\sigma_{1}-\sigma_{2}\right)}^{2}+{\left(\sigma_{2}-\sigma_{3}\right)}^{2}+{\left(\sigma_{1}-\sigma_{3}\right)}^{2}}{2}},$$

where $\sigma_{1}$ is the axial stress caused by bending moments and tensile force, $\sigma_{2}$ is the radial stress, and $\sigma_{3}$ is the hoop stress.

The maximum von Mises stress occurs for the outer-diameter stress of the pipeline and at the position farthest from the bending-neutral layer. Among the three stress components, the axial stress plays a dominant role. Therefore, the position where the maximum value of the axial stress occurs is also the position where the maximum von Mises stress occurs. At this position, the shear stress is 0, and the radial and hoop stresses are provided by the pressures inside and outside the pipeline. The axial, radial, and hoop stresses can be written as the following forms:

(22) $$\sigma_{1}=\frac{N}{a_{stress}}+\frac{M}{2I}OD_{stress},$$

(23) $$a_{stress}=\frac{\pi }{4}(O{D^{2}}_{stress}-I{D^{2}}_{stress}),$$

(24) $$I=\frac{\pi }{64}(O{D^{4}}_{stress}-I{D^{4}}_{stress}),$$

(25) $$\sigma_{2}=\sigma_{RR},$$

(26) $$\sigma_{3}=\sigma_{CC},$$

where *I* is the cross-sectional moment of inertia of the pipeline, and $\sigma_{RR}$ and $\sigma_{CC}$ are the radial and hoop stresses by the internal and external pressures of the pipeline. They are calculated using Lamé’s equation for thick-walled cylinders as

(27) $$\sigma_{RR}=\xi -\frac{\mu^{2}}{O{D^{2}}_{stress}},$$

(28) $$\sigma_{CC}=\xi +\frac{\mu^{2}}{O{D^{2}}_{stress}},$$

(29) $$\xi -\frac{\mu }{{\left(0.5ID_{stress}\right)}^{2}}=-p_{i},\;\xi -\frac{\mu }{{\left(0.5OD_{stress}\right)}^{2}}=-p_{0},$$

where *OD_stress_* and *ID_stress_* are the outer and inner diameters of stress, and *p_i_* and *p*_0_ are the internal and external pressures.

The bending moment and axial force of each point on the pipeline calculated by Equations (11)–(16), as well as the internal and external pressures of the pipeline, are substituted into Equations (21)–(29), then the distribution of the maximum von Mises stress on the pipeline can be calculated.

#### 2.4.2. Load-Controlled Condition (LCC)

According to the Offshore Standard DNVGL-ST-F101 [44], the load-controlled condition (LCC) is a combined loading criterion of local buckling in which the structural response is primarily governed by the imposed loads. In this condition, pipelines subjected to a bending moment, effective axial force, and external overpressure must satisfy a criterion at all cross sections. This criterion states that the calculated value of the LCC cannot be greater than 1. The LCC is expressed as

(30) $$LCC={\left[\gamma_{m}\gamma_{sc}\frac{\left|M\right|}{\alpha_{C}(\alpha_{pm}M_{P})}+{\left(\frac{\gamma_{m}\gamma_{sc}N}{\alpha_{C}S_{P}}\right)}^{2}\right]}^{2}+{\left(\gamma_{m}\gamma_{sc}\frac{p_{0}-p_{\min}}{p_{c}}\right)}^{2},$$

(31) $$\alpha_{C}=(1-\beta )+\beta \frac{f_{u}}{f_{y}},$$

(32) $$\beta =\frac{1}{90}(60-\frac{D}{t_{1}}),$$

(33) $$M_{P}=f_{y}{\left(OD-t_{1}\right)}^{2}t_{1},$$

(34) $$S_{P}=f_{y}(D-t_{1})t_{1},$$

(35) $$(p_{c}-p_{el})({p_{c}}^{2}-{p_{p}}^{2})=p_{c}p_{el}p_{p}f_{0}\frac{D}{t_{1}},$$

(36) $$p_{p}=2f_{y}\alpha_{fab}(\frac{t_{1}}{D}),$$

(37) $$p_{el}=2E{\left(\frac{t_{1}}{D}\right)}^{3}\left(\frac{1}{1-\upsilon^{2}}\right),$$

where *M* is the bending moment at the cross section, *N* is the axial force at the cross section, *t*_1_ is the pipe wall thickness, *f_y_* is the yield stress to be used in design, *p_min_* is the minimum internal pressure, *f_u_* is the tensile strength to be used in design, υ *i*s Poisson’s ratio, *f*_0_ is the ovality, *γ_m_* is the material resistance factor, *γ_sc_* is the safety class resistance factor, *α_fab_* is the fabrication factor, and *α_pm_* is the plastic moment reduction factor for point loads.

The bending moment and axial force at each point of the pipeline determined by Equations (11)–(16) are substituted into Equation (30) to calculate the LCC value for the entire pipeline.

## 3. Model Validation and Parameter Analysis

### 3.1. Model Validation

Comparisons are performed with the results obtained from the present analytical method and the FEM used by Orcaflex to verify the reliability of the analytical model. The basic parameters of the submarine pipeline and the factors of the LCC are shown in Table 1 and Table 2, respectively. The calculated object is a steel pipeline with large diameter and concrete weight coating.

Orcaflex employs a finite element model to represent a line [52]. This model divides the line into multiple segments, each consisting of a straight massless model segment with two nodes at both ends. The model segments are solely responsible for representing the axial and torsional properties of the line. Other properties, such as mass, weight, and buoyancy, are combined at the nodes.

**Figure 5:**
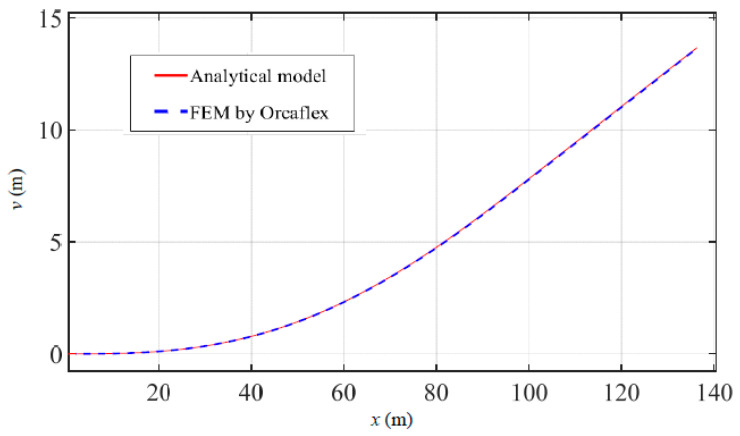
Comparison of pipeline displacements obtained by the present method and the FEM.

**Figure 6:**
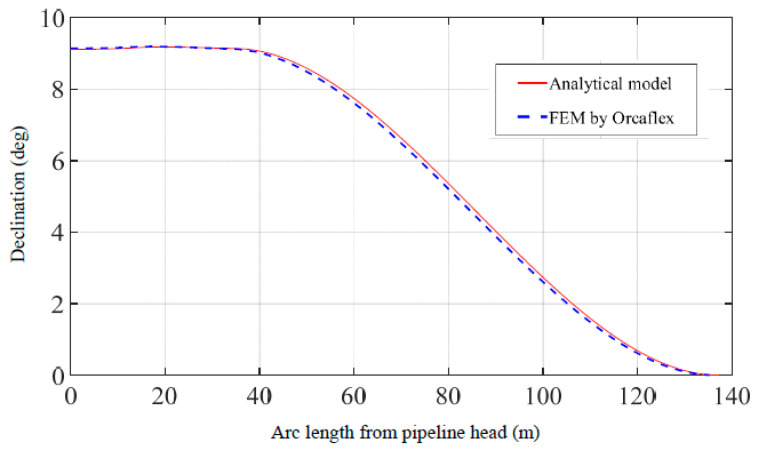
Comparison of pipeline declinations obtained by the present method and the FEM.

**Figure 7:**
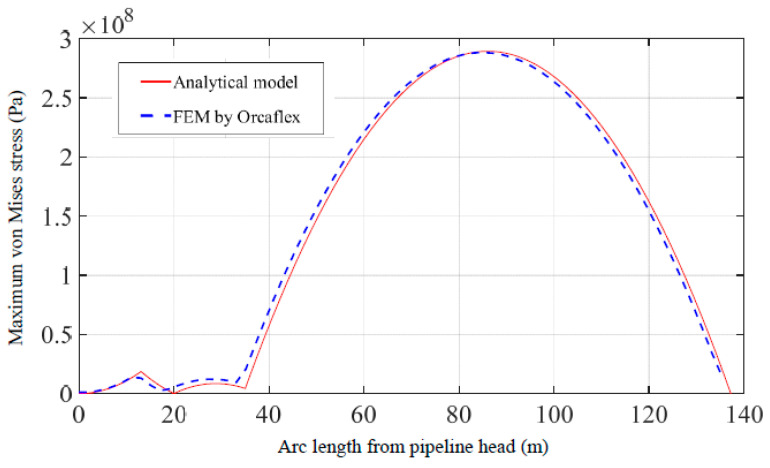
Comparison of maximum von Mises stress of pipeline obtained by the present method and the FEM.

**Figure 8:**
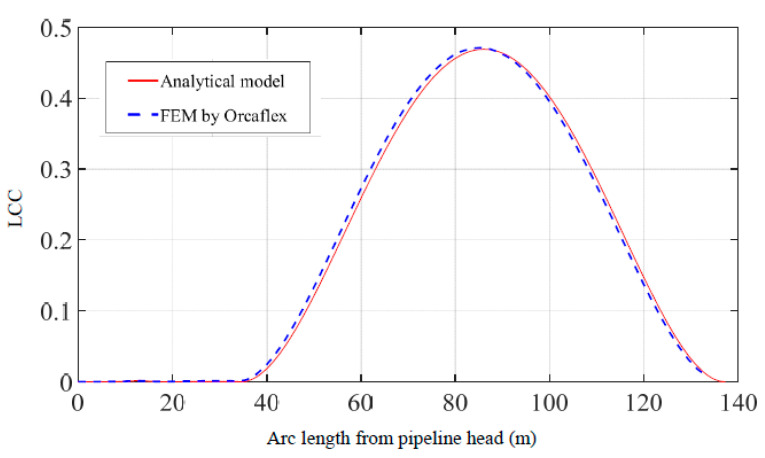
Comparison of pipeline LCC obtained by the present method and the FEM.

According to the multi-point submarine pipeline suspension as shown in Figure 4, in a marine environment with a current speed of 1 m/s, Force 1 (200 kN) is applied at point 1 which is 13 m from the pipeline head, and Force 2 (400 kN) is applied at point 2 which is 35 m from the pipeline head. Comparisons of pipeline displacements, declinations, maximum von Mises stresses, and LCC values are shown in Figure 5, Figure 6, Figure 7 and Figure 8. The results and differences between the analytical model and the FEM used by Orcaflex are shown in Table 3.

As shown in Figure 5, *v* and *x* are the transverse displacement of the pipeline and the horizontal coordinate. Figure 5 shows the pipeline displacements for multi-point submarine pipeline suspension obtained by the present method and the FEM. The transverse displacements of the pipeline appear to be in excellent agreement regarding the results obtained by the two methods. The lengths of the lifting part obtained from the analytical model and the FEM used by Orcaflex are 137.2 m and 134.3 m, respectively, with a difference of 2.1%. Similarly, the displacements of the pipeline head obtained from the two methods are 13.6 m and 13.4 m, with a difference of 1.5%. Figure 6 displays the comparison of the declinations of the entire suspended pipeline, which shows a very close agreement between the two methods. Additionally, as illustrated in Figure 5 and Figure 6, the shapes of the entire pipeline obtained from the analytical model and the FEM used by Orcaflex appear to be in excellent consistency.

The distribution of the maximum von Mises stress along the entire suspended pipeline is illustrated in Figure 7, which shows good agreement among results obtained by the two methods. The maximum stress values obtained from both methods are located at the same position of the pipeline, at 289.5 × 10^6^ Pa and 288.4 × 10^6^ Pa. There are significant changes in maximum von Mises stresses at point 1 and point 2, and the maximum value of maximum stress occurs in the suspended section between point 2 and the TDP.

Furthermore, Figure 8 represents a comparison of LCC values for the entire suspended pipeline calculated using the two methods, demonstrating a strong agreement between the two methods. The maximum values calculated by both methods are 0.469 and 0.472. From Figure 7 and Figure 8, it can be observed that although the distribution laws of the maximum von Mises stress and the LCC along the pipeline are different, their maximum values occur at the same location. Therefore, special attention must be paid to this position during the actual construction process to prevent it from exceeding allowable values.

Table 3 presents the length of lifting part, the height of the pipeline head, the declination of the pipeline head, the peak value of the maximum von Mises stress, and the peak LCC value for the same operating condition using both the present analytical method and the FEM implemented by Orcaflex. It clearly shows that the results obtained from the two methods are consistent.

### 3.2. Effect of Current Velocity

This study focuses on the influence of current velocity on a single-point submarine pipeline suspension as shown in Figure 3. Firstly, Morison’s formulas, Equations (18) and (19), are used to establish the relationship between ocean current velocity and the environmental loads acting on the pipeline. The pipeline model shown in Table 1 is then used as the research object. In order to compare the displacements and mechanical properties of the pipeline under different current velocities, a force of 500 kN is applied at point 1 which is 13 m from the pipeline head.

**Figure 9:**
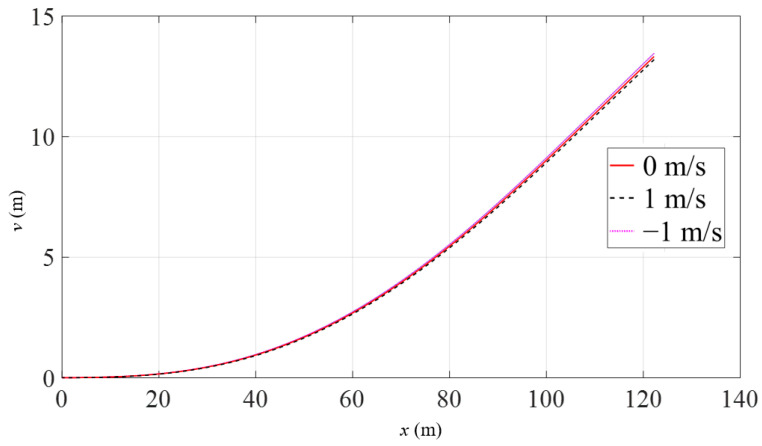
Pipeline displacements with the variation of current velocity.

As shown in Figure 9, there is little difference in pipeline displacements across different current velocities. However, the force of the current has an upward lifting effect on the pipeline when the angle between the current velocity and the axis of the pipeline is obtuse, while it has a downward effect when the angle is acute. Despite the large weight and flexural rigidity of the pipeline, the effect of ocean currents is subtle.

**Figure 10:**
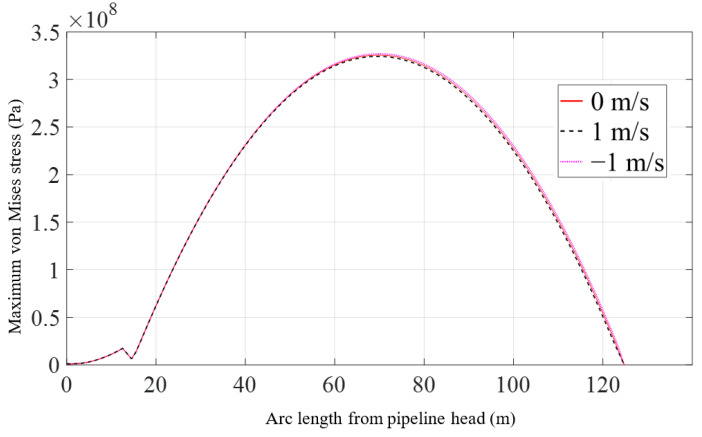
Maximum von Mises stress of the pipeline with the variation of current velocity.

**Figure 11:**
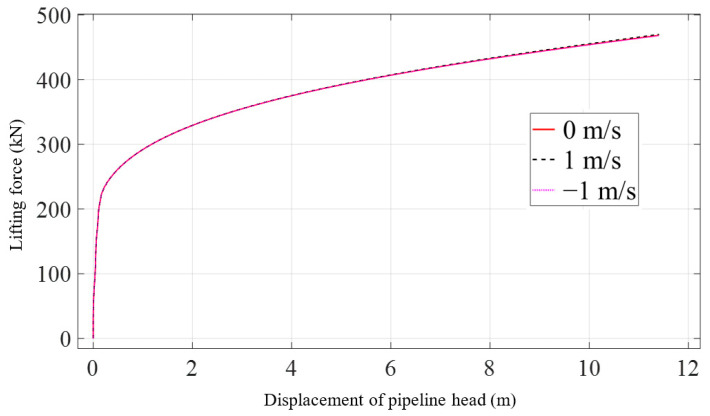
Lifting force with the variation of current velocity.

Figure 10 shows that there is also little difference in the peak values of the maximum von Mises stress of the pipeline across different current velocities. These peak values are consistent and occur at the same locations. Figure 11 illustrates the relationship between the displacement of the pipeline head and the lifting force under conditions of three current velocities. At the beginning of lifting, the force increases rapidly as the displacement changes, but thereafter, the change becomes slow.

### 3.3. Effect of Lifting Point Position

The position of the lifting point plays a significant role in submarine pipeline suspension. In this section, the influence of the distance between the lifting point and pipeline head on the lifting process of the pipeline is studied. The research object is the pipeline model with large diameter as shown in Table 1. Single-point submarine pipeline suspension is considered in order to show the differences clearly.

The specific scheme is set as a single lifting point located at 0 m, 4 m, or 12 m away from the pipeline head. Under three different construction configurations, the pipeline head is lifted to the same position. The differences in pipeline shape and mechanical properties when the pipeline head is lifted to the same height are observed, and then the influence of lifting point position on the lifting process of the pipeline is studied.

**Figure 12:**
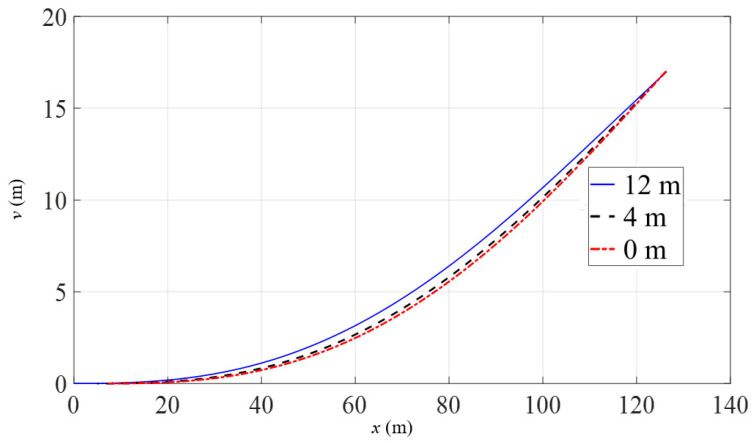
Pipeline displacement with the variation of lifting point position.

**Figure 13:**
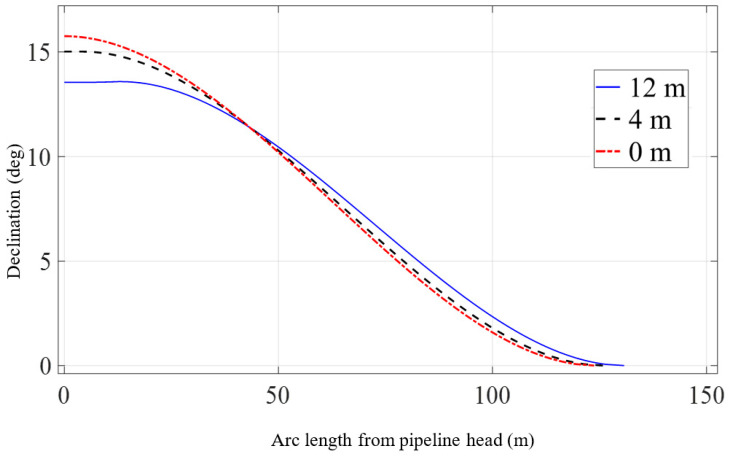
Pipeline declination with the variation of lifting point position.

Figure 12 and Figure 13 show the displacements and declinations of the pipeline under three different conditions when the pipeline head is lifted to a height of 17 m. From Figure 12, it can be observed that as the lifting point moves further away from the pipeline head, the pipeline shape becomes smoother, but the length of the suspended section slightly increases. However, due to the high bending stiffness of the pipeline, there is no significant difference in overall shape. From Figure 13, it is evident that when the lifting point is 12 m away from the pipeline head, the declination of the pipeline head is significantly smaller compared to the situation when the suspension point is closer to the pipeline head. The further the lifting point is from the pipeline head, the smaller the declination of the pipeline head for the same lifting height. The declinations between the lifting point and the pipeline head are mostly consistent, indicating that this section does not undergo noticeable bending deformation.

**Figure 14:**
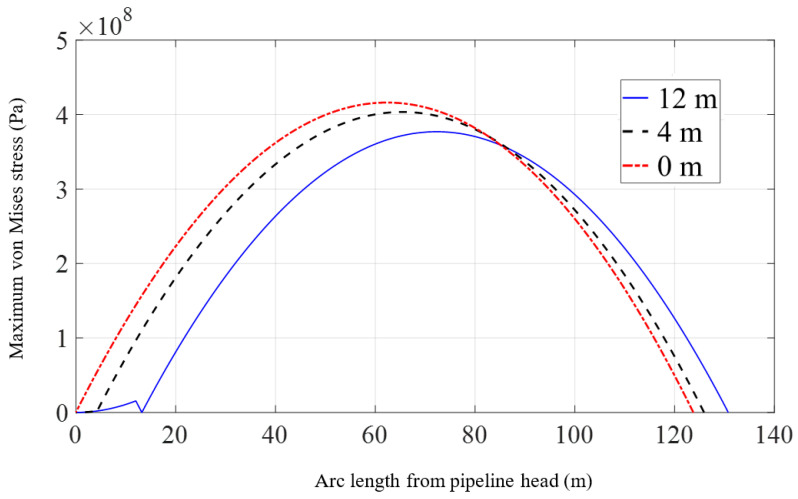
Maximum von Mises stress of the pipeline with the variation of lifting point position.

Figure 14 exhibits the distributions of the maximum von Mises stress in the pipeline under three different scenarios when the elevation of the pipeline head is increased to 17 m. In comparison to the geometric changes, the location of the lifting point has a more noticeable impact on the distribution of the maximum von Mises stress along the pipeline. It is worth noting that according to the preliminary simplified criteria for local buckling checks in the early design stage provided by DNVGL-ST-F101 [44], the pipeline stress verification requires that the maximum von Mises stress of the pipeline should be less than 0.87 *f_y_*. For the model in this study, the allowable stress is 389.8 × 10^6^ Pa. However, when the lifting point is located near the pipeline head and the pipeline is lifted by 17 m, the maximum stress of the pipeline is 416.1 × 10^6^ Pa, which exceeds the requirement of DNV [44]. As the distance between the lifting point and the pipeline head increases, the maximum value of the maximum von Mises stress on the pipeline decreases, and its occurrence shifts away from the pipeline head. When the lifting point is adjusted from the pipeline head to a distance of 12 m from the pipeline head, the maximum value of the maximum von Mises stress in the suspended section of the pipeline decreases from 416.1 × 10^6^ Pa to 376.9 × 10^6^ Pa, indicating a 9.5% decrease in stress amplitude. At this point, the overall stress distribution of the pipeline is within the allowable range specified by DNV [44]. Furthermore, the location of the maximum value moves from 62.4 m to 72.3 m away from the head.

**Figure 15:**
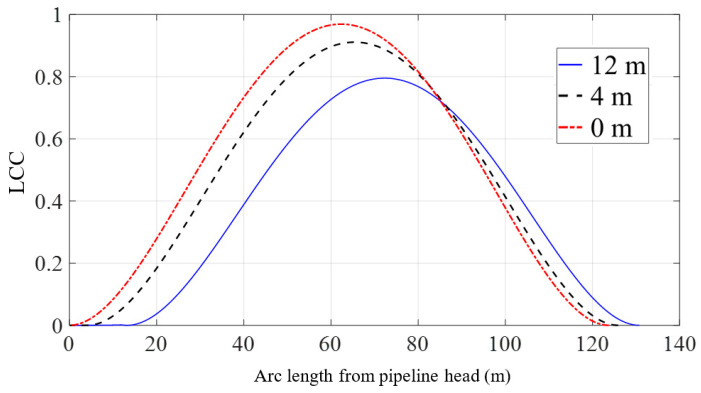
LCC of the pipeline with the variation of lifting point position.

Figure 15 illustrates the distribution of the LCC in the suspended section of the pipeline under the three different scenarios. From the figure, it can be observed that when the lifting point is at a distance of 12 m from the pipeline head, the maximum value of the LCC is 0.79, which is 18.6% lower than the maximum value of 0.97 when the lifting point is at the pipeline head. All values calculated for the LCC meet the standard set by DNV, which requires the LCC to be lower than 1. A conclusion can be drawn that adjusting the position of the lifting point appropriately decreases the maximum value of the maximum von Mises stress on the pipeline and enhances the safety of the pipeline-lifting operation.

**Figure 16:**
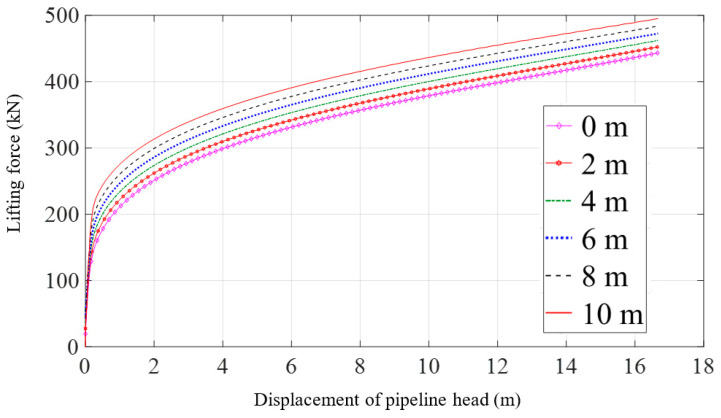
Lifting force with the variation of displacement of pipeline head under different lifting point positions.

**Figure 17:**
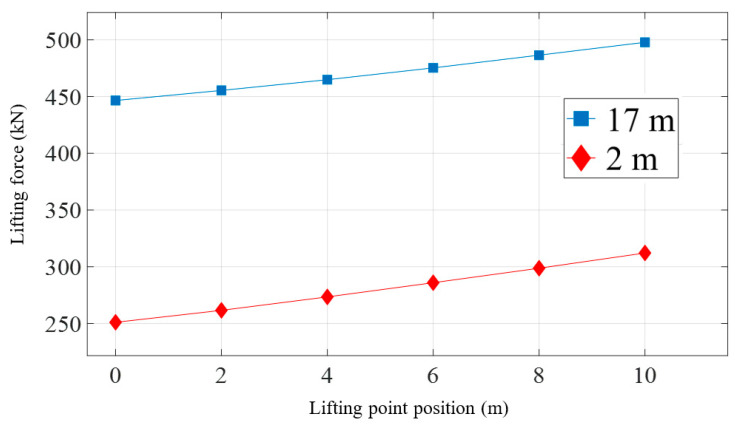
Lifting force with the variation of lifting point position under the same displacement of pipeline head.

After conducting a static analysis on three different positions of the lifting point that results in a 17 m height of the pipeline head, an analysis of the lifting process for different lifting point positions is performed. Figure 16 presents the curves depicting the variation of the lifting force with the lifting height for different lifting point positions while lifting the pipeline head to 17 m. The lifting point position differs from the pipeline head location up to a distance of 10 m. It can be observed that during the initial stage of lifting when the pipeline is about to be raised, the lifting point position has little influence on the lifting force, as the curves almost overlap at this stage. However, as the pipeline is gradually lifted, the farther the lifting point is from the pipeline head, the greater the required lifting force, as visually shown in Figure 17. Specifically, when the pipeline head is lifted to 2 m, adjusting the lifting point from the pipeline head to a distance of 10 m results in an increase in the lifting force from 251.1 kN to 312.2 kN. Similarly, when the pipeline head is lifted to 17 m, adjusting the lifting point from the pipeline head to a distance of 10 m results in an increase in the lifting force from 446.6 kN to 497.9 kN.

**Figure 18:**
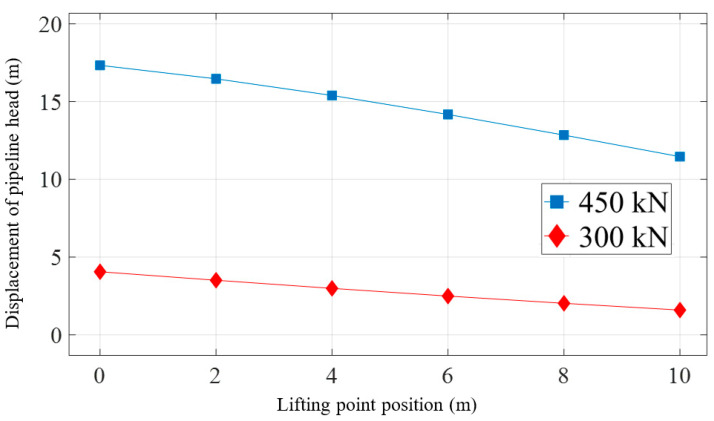
Displacement of the pipeline head with the variation of lifting point position under the same force.

Figure 18 represents the variation of the head height with the lifting point position under the same lifting force. When the force is 300 kN, adjusting the lifting point from the pipeline head to a distance of 10 m leads to a decrease in the pipeline head height from 4.1 m to 1.6 m, a reduction of 61%. In the meantime, when the force is 450 kN, the pipeline head height decreases from 17.3 m to 11.5 m, which corresponds to a reduction of 34%.

## 4. Conclusions

This study establishes an analysis model for pipeline lifting during an offshore pipeline installation process. The boundary problems are converted into initial value problems using the shooting and secant methods. The Runge–Kutta method is then employed to solve the differential equations for the displacement and mechanical performance of the pipeline. The mechanical properties of the submarine pipeline are further verified and checked according to the Det Norske Veritas (DNV) standard which ensures the safety of offshore engineering. To validate the accuracy of the theoretical calculations, Orcaflex is used, and our results show good agreement with the FEM results from Orcaflex, indicating the accuracy of the proposed model in determining the shape and mechanical properties of the pipeline during the offshore pipeline installation process.

Moreover, investigations are conducted to evaluate the impact of factors such as ocean current velocity and lifting position on the pipe-lifting process. The results indicate that ocean current velocity has minimal influence on the pipe shape and mechanical performance during the pipeline-lifting process. The shape and mechanical properties of the pipe exhibit significant changes with variations in the lifting position. When analyzing the shape of the pipeline, it is observed that the length of the suspended section of the pipeline increases as the lifting point moves farther away from the pipeline head when lifting the pipeline head to the same height and results in a reduction in the declination of the pipeline head, leading to a decrease in the overall bending deformation of the pipeline. Additionally, under the same scenario related to the mechanical properties of the pipeline, both the peak value of the LCC and the maximum von Mises stress of the pipeline significantly decrease as the lifting point moves further away from the pipeline head. Furthermore, the position of the peak value also moves away from the pipeline head, despite an increase in the lifting force. Moreover, when considering the same lifting force, the farther the lifting point is from the pipeline head, the lower the height is at the pipeline head. Repositioning the lifting point on the pipeline can ensure that the mechanical properties of the pipeline meet the requirements of construction standards, thereby serving as a reference for actual construction processes.

In conclusion, the analysis of the proposed model verifies its ability to predict the mechanical behavior of offshore pipeline lifting. This study provides reasonable guidance for the application of pipeline lifting in offshore engineering. Nonetheless, further research is required to investigate the dynamic response of pipelines during the lifting process.

## Figures and Tables

**Table 1:** The parameters of pipeline.

Descriptions	Values
Structural material	Steel
Classification	X65
Outer diameter (m)	1.2
Wall thickness (m)	0.03
Density (kg/m^3^)	7850
Young’s modulus (Pa)	2.07 × 10^11^
Poisson’s ratio	0.3
Yield stress (Pa)	448 × 10^6^
Tensile strength (Pa)	531 × 10^6^
Weight coating material	Concrete
Density of weight coating (kg/m^3^)	3044
Thickness of weight coating (m)	0.12
Content	Air

**Table 2:** The factors of LCC.

Descriptions	Value
Ovality, $f_{0}$	0
Material resistance factor, $\gamma_{m}$	1.15
Safety class resistance factor, $\gamma_{sc}$	1.26
Fabrication factor, $\alpha_{fab}$	0.93
Plastic moment reduction factor, $\alpha_{pm}$	1

**Table 3:** Result comparisons of analytical model and FEM used by Orcaflex.

Descriptions	Analytical Model	FEM Used by Orcaflex	Relative Error (%)
Length of lifting part (m)	137.2	134.3	2.2
Height of pipeline head (m)	13.6	13.4	1.5
Declination of pipeline head (deg)	9.0	9.1	1.1
Peak value of maximum von Mises stress (Pa)	289.5 × 10^6^	288.4 × 10^6^	0.4
Peak value of LCC	0.469	0.472	0.6

## Data Availability

Data available on request due to restrictions eg privacy or ethical. The data presented in this study are available on request from the corresponding author.

## Abbreviations/Nomenclature

*a*	length of the suspended section of the pipeline
*C_n_*	normal drag coefficient
*C_t_*	tangential drag coefficient
*D*	outer diameter of the steel part of the pipeline
*E*	Young’s modulus
*EI*	flexural rigidity of the pipeline
*F_n_*	normal environmental loading in the section of pipeline
*F_t_*	tangential environmental loading in the section of pipeline
*f*_0_	ovality
*f_u_*	tensile strength to be used in design
*f_y_*	yield stress to be used in design
*ID_stress_*	inner diameter of stress
*M*	bending moment of the section of pipeline
*N*	axial force in the section of pipeline
*OD_stress_*	outer diameter of stress
*p*_0_	external pressure
*p_i_*	internal pressure
*p_min_*	minimum internal pressure
*q*	gravity of unit length of the pipeline in the sea
*Q*	shear force in the section of pipeline
*s*	length of the pipeline
*t_1_*	pipeline wall thickness
*t_2_*	thickness of coating layer
*u*	transverse displacement of the pipeline
*v*	axial displacement of the pipeline
*V_c_*	velocity of ocean current
*α_fab_*	fabrication factor
*α_pm_*	plastic moment reduction factor for point loads
*γ_m_*	material resistance factor
*γ_sc_*	safety class resistance factor
*ρ*_s_	density of seawater
*θ*	inclination angle of the section of pipeline
*σ*_1_	axial stress
*σ*_2_	radial stress
*σ*_3_	hoop stress
